# 2-(4-Chloro­anilino)-3-(2-hydroxy­ethyl)quinazolin-4(3*H*)-one

**DOI:** 10.1107/S1600536808036623

**Published:** 2008-11-13

**Authors:** Hong-Ling Wang, Xu-Hong Yang, Ming-Hu Wu

**Affiliations:** a Faculty of Chemistry and Life Science, Xianning University, Xianning 437100, People’s Republic of China

## Abstract

In the title mol­ecule, C_16_H_14_ClN_3_O_2_, the dihedral angle between the chloro­phenyl and pyrimidinone rings is 14.8 (1)°, while the dihedral angle between the fused benzene ring and the pyrimidinone ring is 3.8 (1)°. In the crystal structure, intra­molecular N—H⋯O hydrogen bonds, together with inter­molecular O—H⋯O hydrogen-bonding inter­actions, are present.

## Related literature

For the biological activities and applications of 4(3*H*)-quinazolinone, see: Armarego (1963 ▶); Fisnerova *et al.* (1986 ▶); Gravier *et al.* (1992 ▶). For details of our ongoing heterocyclic synthesis and drug discovery project, see: Yang *et al.* (2008 ▶).
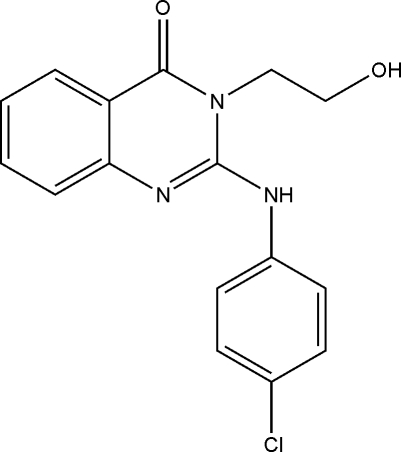


## Experimental

### 

#### Crystal data

C_16_H_14_ClN_3_O_2_
*M* *_r_* = 315.75
Monoclinic, 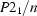

*a* = 9.0707 (18) Å
*b* = 11.345 (2) Å
*c* = 14.143 (3) Å
β = 96.98 (3)°
*V* = 1444.6 (5) Å^3^
*Z* = 4
Mo *K*α radiation
μ = 0.28 mm^−1^
*T* = 273 (2) K
0.20 × 0.20 × 0.10 mm

#### Data collection

Bruker SMART APEX CCD diffractometer
Absorption correction: multi-scan (*SADABS*; Sheldrick, 2001 ▶) *T*_min_ = 0.947, *T*_max_ = 0.973
8113 measured reflections
2824 independent reflections
2300 reflections with *I* > 2σ(*I*)
*R*_int_ = 0.019

#### Refinement

*R*[*F* ^2^ > 2σ(*F* ^2^)] = 0.037
*wR*(*F* ^2^) = 0.108
*S* = 1.05
2824 reflections
205 parameters
H atoms treated by a mixture of independent and constrained refinement
Δρ_max_ = 0.16 e Å^−3^
Δρ_min_ = −0.24 e Å^−3^

### 

Data collection: *SMART* (Bruker, 2001 ▶); cell refinement: *SAINT-Plus* (Bruker, 2001 ▶; data reduction: *SAINT-Plus*; program(s) used to solve structure: *SHELXS97* (Sheldrick, 2008 ▶); program(s) used to refine structure: *SHELXL97* (Sheldrick, 2008 ▶); molecular graphics: *PLATON* (Spek, 2003 ▶); software used to prepare material for publication: *PLATON*.

## Supplementary Material

Crystal structure: contains datablocks global, I. DOI: 10.1107/S1600536808036623/ez2147sup1.cif

Structure factors: contains datablocks I. DOI: 10.1107/S1600536808036623/ez2147Isup2.hkl

Additional supplementary materials:  crystallographic information; 3D view; checkCIF report

## Figures and Tables

**Table 1:** Hydrogen-bond geometry (Å, °)

*D*—H⋯*A*	*D*—H	H⋯*A*	*D*⋯*A*	*D*—H⋯*A*
N1—H1*A*⋯O2	0.839 (18)	1.993 (19)	2.8017 (19)	161.8 (17)
O2—H2*A*⋯O1^i^	0.86 (2)	1.86 (2)	2.7180 (18)	174 (2)

Symmetry code: (i) 
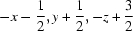
.

**Fig. 1.**
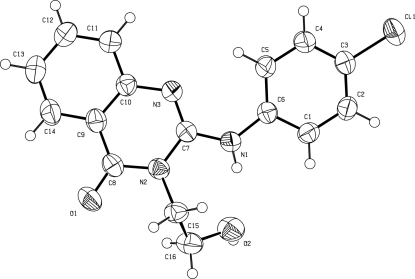
View of the molecule with the atom-labeling scheme. Displacement ellipsoids are drawn at the 50% probability level.

**Fig. 2.**
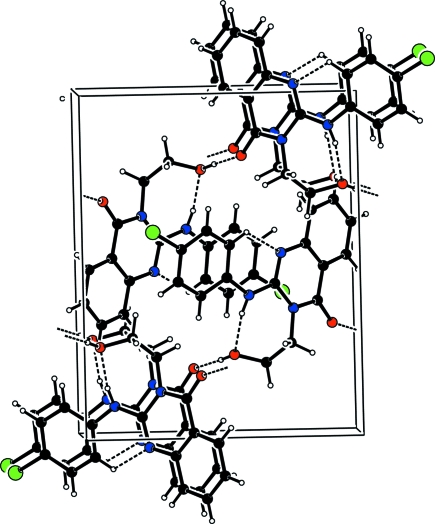
Crystal packing viewed down the a axis. Hydrogen bonds are shown as dashed lines.

## supplementary crystallographic information

### Comment

One of the most frequently encountered heterocyclic molecules in medicinal chemistry is 4(3*H*)-quinazolinone, which has wide application as a result of antibacterial, antifungal, anticonvulsant, and anti-inflammatory activities (Armarego, 1963; Gravier *et al.*, 1992; Fisnerova *et al.*, 1986). In our ongoing heterocyclic synthesis and drug discovery project (Yang *et al.*, 2008) we have focused on the synthesis of quinazolinones and pyrazolo pyrimidinones. Herein, the title compound was synthesized and determined by single-crystal X-ray diffraction.

In the molecule (Fig. 1), the dihedral angle between the chlorophenyl and pyrimidinone rings is 14.8 (1)°, and the dihedral angle between the fused benzene and pyrimidinone rings is 3.8 (1)°.

In the crystal structure, molecules are linked by intramolecular N1–H1A···O2 hydrogen-bonds together with O2–H2A···O1^i^ intermolecular hydrogen-bonding interactions (symmetry code: i, -1/2 - *x*,1/2 + *y*,3/2 - *z*) (Fig. 2).

### Experimental

To a solution of 2-ethoxycarbonyliminophosphorane (1.27 g, 3 mmol) in 10 ml absolute anhydrous CH_2_Cl_2_, 4-chlorophenylisocyanate (0.46 g, 3 mmol) was added dropwise at room temperature. The reaction mixture was left unstirred for 6 h at 273–278 K, whereafter a solution of 2-hydroxyethylamine (0.18 g, 3 mmol) in 5 ml absolute anhydrous CH_2_Cl_2_was added. The reaction mixture was then stirred overnight, the solution cooled and the reaction product recrystallized from CH_3_OH to give colorless crystals of the title compound suitable for X-ray analysis in 58% yield.

### Refinement

H atoms bonded to C atoms were placed in calculated positions (C—H = 0.93–0.97 Å) and included in the riding model approximation. The positional parameters of H atoms bonded to N and O atoms were refined independently. For all H atoms *U*_iso_ (H) = 1.2*U*_iso_ (C,*N*) or 1.5*U*_iso_ (O).

### Crystal data

**Table:**

C_16_H_14_ClN_3_O_2_	*F*_000_ = 656
*M**_r_* = 315.75	*D*_x_ = 1.452 Mg m^−^^3^
Monoclinic, *P*2_1_/*n*	Melting point = 432–434 K
Hall symbol: -P 2yn	Mo *K*α radiation λ = 0.71073 Å
*a* = 9.0707 (18) Å	Cell parameters from 3170 reflections
*b* = 11.345 (2) Å	θ = 2.3–26.2º
*c* = 14.143 (3) Å	µ = 0.28 mm^−^^1^
β = 96.98 (3)º	*T* = 273 (2) K
*V* = 1444.6 (5) Å^3^	Block, colourless
*Z* = 4	0.20 × 0.20 × 0.10 mm

### Data collection

**Table:**

Bruker SMART APEX CCD diffractometer	2824 independent reflections
Radiation source: fine-focus sealed tube	2300 reflections with *I* > 2σ(*I*)
Monochromator: graphite	*R*_int_ = 0.019
*T* = 273(2) K	θ_max_ = 26.0º
φ and ω scans	θ_min_ = 2.3º
Absorption correction: multi-scan(SADABS; Sheldrick, 2001)	*h* = −11→9
*T*_min_ = 0.947, *T*_max_ = 0.973	*k* = −11→13
8113 measured reflections	*l* = −17→17

### Refinement

**Table:**

Refinement on *F*^2^	Secondary atom site location: difference Fourier map
Least-squares matrix: full	Hydrogen site location: inferred from neighbouring sites
*R*[*F*^2^ > 2σ(*F*^2^)] = 0.037	H atoms treated by a mixture of independent and constrained refinement
*wR*(*F*^2^) = 0.108	*w* = 1/[σ^2^(*F*_o_^2^) + (0.0587*P*)^2^ + 0.2177*P*] where *P* = (*F*_o_^2^ + 2*F*_c_^2^)/3
*S* = 1.05	(Δ/σ)_max_ = 0.001
2824 reflections	Δρ_max_ = 0.16 e Å^−^^3^
205 parameters	Δρ_min_ = −0.24 e Å^−^^3^
Primary atom site location: structure-invariant direct methods	Extinction correction: none

### Special details

**Table:**

Geometry. All e.s.d.'s (except the e.s.d. in the dihedral angle between two l.s. planes) are estimated using the full covariance matrix. The cell e.s.d.'s are taken into account individually in the estimation of e.s.d.'s in distances, angles and torsion angles; correlations between e.s.d.'s in cell parameters are only used when they are defined by crystal symmetry. An approximate (isotropic) treatment of cell e.s.d.'s is used for estimating e.s.d.'s involving l.s. planes.
Refinement. Refinement of *F*^2^ against ALL reflections. The weighted *R*-factor *wR* and goodness of fit *S* are based on *F*^2^, conventional *R*-factors *R* are based on *F*, with *F* set to zero for negative *F*^2^. The threshold expression of *F*^2^ > σ(*F*^2^) is used only for calculating *R*-factors(gt) *etc*. and is not relevant to the choice of reflections for refinement. *R*-factors based on *F*^2^ are statistically about twice as large as those based on *F*, and *R*- factors based on ALL data will be even larger.

### Fractional atomic coordinates and isotropic or equivalent isotropic displacement parameters (Å^2^)

**Table:**

	*x*	*y*	*z*	*U*_iso_*/*U*_eq_	
C1	0.24608 (18)	1.06958 (14)	0.90802 (11)	0.0460 (4)	
H1	0.2103	1.0780	0.8439	0.055*	
C2	0.34154 (18)	1.15287 (15)	0.95164 (11)	0.0495 (4)	
H2	0.3699	1.2173	0.9174	0.059*	
C3	0.39474 (17)	1.13990 (14)	1.04660 (12)	0.0460 (4)	
C4	0.35073 (19)	1.04650 (15)	1.09827 (12)	0.0524 (4)	
H4	0.3857	1.0395	1.1626	0.063*	
C5	0.25416 (18)	0.96263 (15)	1.05448 (11)	0.0502 (4)	
H5	0.2242	0.8995	1.0895	0.060*	
C6	0.20225 (16)	0.97305 (13)	0.95836 (11)	0.0397 (3)	
C7	0.03780 (15)	0.79644 (13)	0.93558 (10)	0.0396 (3)	
C8	−0.13344 (17)	0.63934 (14)	0.88093 (11)	0.0465 (4)	
C9	−0.13969 (17)	0.61551 (14)	0.98083 (11)	0.0460 (4)	
C10	−0.04823 (16)	0.68016 (14)	1.04878 (11)	0.0427 (4)	
C11	−0.05282 (18)	0.65638 (16)	1.14562 (12)	0.0520 (4)	
H11	0.0091	0.6974	1.1915	0.062*	
C12	−0.1479 (2)	0.57314 (17)	1.17282 (13)	0.0608 (5)	
H12	−0.1512	0.5588	1.2373	0.073*	
C13	−0.2398 (2)	0.50952 (18)	1.10526 (15)	0.0672 (5)	
H13	−0.3044	0.4533	1.1247	0.081*	
C14	−0.2352 (2)	0.52964 (16)	1.01032 (13)	0.0607 (5)	
H14	−0.2956	0.4862	0.9652	0.073*	
C15	−0.00780 (19)	0.74122 (15)	0.76189 (11)	0.0477 (4)	
H15A	−0.0208	0.6648	0.7312	0.057*	
H15B	0.0951	0.7643	0.7613	0.057*	
C16	−0.1053 (2)	0.82925 (16)	0.70402 (12)	0.0572 (4)	
H16A	−0.1005	0.8163	0.6367	0.069*	
H16B	−0.2076	0.8194	0.7161	0.069*	
Cl1	0.51722 (6)	1.24474 (4)	1.10084 (4)	0.06868 (19)	
N1	0.10834 (15)	0.89234 (12)	0.90499 (9)	0.0450 (3)	
H1A	0.0750 (19)	0.9182 (16)	0.8510 (13)	0.054*	
N2	−0.03685 (14)	0.72897 (11)	0.86188 (9)	0.0422 (3)	
N3	0.03988 (14)	0.77194 (11)	1.02486 (9)	0.0436 (3)	
O1	−0.20317 (14)	0.58621 (11)	0.81420 (9)	0.0639 (4)	
O2	−0.05636 (14)	0.94472 (12)	0.72940 (9)	0.0606 (3)	
H2A	−0.129 (3)	0.992 (2)	0.7132 (17)	0.091*	

### Atomic displacement parameters (Å^2^)

**Table:**

	*U*^11^	*U*^22^	*U*^33^	*U*^12^	*U*^13^	*U*^23^
C1	0.0555 (9)	0.0443 (9)	0.0388 (8)	−0.0021 (7)	0.0088 (7)	0.0014 (7)
C2	0.0578 (10)	0.0417 (9)	0.0510 (9)	−0.0077 (7)	0.0148 (7)	0.0012 (7)
C3	0.0454 (8)	0.0386 (8)	0.0536 (9)	−0.0025 (7)	0.0049 (7)	−0.0039 (7)
C4	0.0591 (10)	0.0470 (9)	0.0480 (9)	−0.0069 (8)	−0.0063 (7)	0.0057 (7)
C5	0.0551 (10)	0.0435 (9)	0.0493 (9)	−0.0087 (7)	−0.0040 (7)	0.0097 (7)
C6	0.0385 (8)	0.0355 (8)	0.0450 (8)	0.0015 (6)	0.0043 (6)	−0.0006 (6)
C7	0.0374 (8)	0.0374 (8)	0.0431 (8)	0.0016 (6)	0.0016 (6)	−0.0023 (6)
C8	0.0476 (9)	0.0403 (9)	0.0512 (9)	−0.0021 (7)	0.0043 (7)	−0.0092 (7)
C9	0.0481 (9)	0.0372 (8)	0.0531 (9)	−0.0018 (7)	0.0078 (7)	−0.0045 (7)
C10	0.0421 (8)	0.0391 (8)	0.0465 (8)	0.0006 (6)	0.0035 (6)	0.0020 (7)
C11	0.0526 (9)	0.0539 (10)	0.0482 (9)	−0.0061 (8)	0.0008 (7)	0.0042 (8)
C12	0.0657 (11)	0.0616 (12)	0.0556 (10)	−0.0088 (9)	0.0093 (9)	0.0124 (9)
C13	0.0748 (13)	0.0568 (12)	0.0714 (12)	−0.0225 (10)	0.0149 (10)	0.0049 (10)
C14	0.0681 (11)	0.0491 (10)	0.0654 (11)	−0.0179 (9)	0.0099 (9)	−0.0083 (9)
C15	0.0560 (10)	0.0468 (9)	0.0403 (8)	0.0042 (7)	0.0065 (7)	−0.0050 (7)
C16	0.0626 (11)	0.0598 (11)	0.0466 (9)	0.0048 (9)	−0.0035 (8)	−0.0017 (8)
Cl1	0.0759 (4)	0.0539 (3)	0.0731 (3)	−0.0218 (2)	−0.0037 (3)	−0.0055 (2)
N1	0.0489 (7)	0.0433 (8)	0.0408 (7)	−0.0053 (6)	−0.0027 (6)	0.0043 (6)
N2	0.0466 (7)	0.0386 (7)	0.0411 (7)	0.0003 (5)	0.0039 (5)	−0.0046 (5)
N3	0.0445 (7)	0.0434 (7)	0.0421 (7)	−0.0048 (5)	0.0018 (5)	0.0005 (6)
O1	0.0715 (8)	0.0628 (8)	0.0562 (7)	−0.0195 (6)	0.0029 (6)	−0.0179 (6)
O2	0.0660 (8)	0.0522 (8)	0.0596 (7)	0.0103 (6)	−0.0079 (6)	0.0050 (6)

### Geometric parameters (Å, °)

**Table:**

C1—C2	1.376 (2)	C9—C14	1.400 (2)
C1—C6	1.390 (2)	C10—N3	1.3796 (19)
C1—H1	0.9300	C10—C11	1.402 (2)
C2—C3	1.379 (2)	C11—C12	1.365 (2)
C2—H2	0.9300	C11—H11	0.9300
C3—C4	1.374 (2)	C12—C13	1.391 (3)
C3—Cl1	1.7408 (16)	C12—H12	0.9300
C4—C5	1.387 (2)	C13—C14	1.368 (3)
C4—H4	0.9300	C13—H13	0.9300
C5—C6	1.389 (2)	C14—H14	0.9300
C5—H5	0.9300	C15—N2	1.476 (2)
C6—N1	1.406 (2)	C15—C16	1.508 (2)
C7—N3	1.2907 (19)	C15—H15A	0.9700
C7—N1	1.3593 (19)	C15—H15B	0.9700
C7—N2	1.400 (2)	C16—O2	1.415 (2)
C8—O1	1.2283 (19)	C16—H16A	0.9700
C8—N2	1.390 (2)	C16—H16B	0.9700
C8—C9	1.446 (2)	N1—H1A	0.839 (18)
C9—C10	1.398 (2)	O2—H2A	0.86 (2)
			
C2—C1—C6	120.99 (15)	C12—C11—H11	119.9
C2—C1—H1	119.5	C10—C11—H11	119.9
C6—C1—H1	119.5	C11—C12—C13	120.77 (17)
C1—C2—C3	119.35 (15)	C11—C12—H12	119.6
C1—C2—H2	120.3	C13—C12—H12	119.6
C3—C2—H2	120.3	C14—C13—C12	120.04 (17)
C4—C3—C2	120.70 (15)	C14—C13—H13	120.0
C4—C3—Cl1	120.26 (13)	C12—C13—H13	120.0
C2—C3—Cl1	119.04 (13)	C13—C14—C9	120.15 (17)
C3—C4—C5	120.01 (15)	C13—C14—H14	119.9
C3—C4—H4	120.0	C9—C14—H14	119.9
C5—C4—H4	120.0	N2—C15—C16	114.90 (14)
C4—C5—C6	119.95 (15)	N2—C15—H15A	108.5
C4—C5—H5	120.0	C16—C15—H15A	108.5
C6—C5—H5	120.0	N2—C15—H15B	108.5
C5—C6—C1	118.97 (14)	C16—C15—H15B	108.5
C5—C6—N1	125.54 (14)	H15A—C15—H15B	107.5
C1—C6—N1	115.48 (14)	O2—C16—C15	109.27 (14)
N3—C7—N1	122.16 (14)	O2—C16—H16A	109.8
N3—C7—N2	123.99 (14)	C15—C16—H16A	109.8
N1—C7—N2	113.85 (13)	O2—C16—H16B	109.8
O1—C8—N2	119.21 (15)	C15—C16—H16B	109.8
O1—C8—C9	125.53 (15)	H16A—C16—H16B	108.3
N2—C8—C9	115.25 (13)	C7—N1—C6	128.91 (13)
C10—C9—C14	119.79 (15)	C7—N1—H1A	116.0 (12)
C10—C9—C8	118.88 (14)	C6—N1—H1A	112.9 (13)
C14—C9—C8	121.33 (15)	C8—N2—C7	121.00 (13)
N3—C10—C9	122.70 (14)	C8—N2—C15	116.40 (13)
N3—C10—C11	118.15 (14)	C7—N2—C15	122.37 (13)
C9—C10—C11	119.01 (14)	C7—N3—C10	117.54 (13)
C12—C11—C10	120.22 (16)	C16—O2—H2A	107.7 (16)
			
C6—C1—C2—C3	0.2 (2)	C12—C13—C14—C9	−1.0 (3)
C1—C2—C3—C4	−1.5 (3)	C10—C9—C14—C13	0.4 (3)
C1—C2—C3—Cl1	179.23 (12)	C8—C9—C14—C13	−179.47 (17)
C2—C3—C4—C5	1.3 (3)	N2—C15—C16—O2	75.96 (19)
Cl1—C3—C4—C5	−179.42 (13)	N3—C7—N1—C6	6.0 (2)
C3—C4—C5—C6	0.1 (3)	N2—C7—N1—C6	−174.53 (14)
C4—C5—C6—C1	−1.4 (2)	C5—C6—N1—C7	7.1 (3)
C4—C5—C6—N1	177.75 (15)	C1—C6—N1—C7	−173.72 (14)
C2—C1—C6—C5	1.2 (2)	O1—C8—N2—C7	176.07 (14)
C2—C1—C6—N1	−178.01 (14)	C9—C8—N2—C7	−5.2 (2)
O1—C8—C9—C10	177.05 (16)	O1—C8—N2—C15	−9.3 (2)
N2—C8—C9—C10	−1.6 (2)	C9—C8—N2—C15	169.47 (13)
O1—C8—C9—C14	−3.1 (3)	N3—C7—N2—C8	9.8 (2)
N2—C8—C9—C14	178.30 (15)	N1—C7—N2—C8	−169.68 (13)
C14—C9—C10—N3	−174.93 (15)	N3—C7—N2—C15	−164.55 (14)
C8—C9—C10—N3	5.0 (2)	N1—C7—N2—C15	16.0 (2)
C14—C9—C10—C11	0.8 (2)	C16—C15—N2—C8	94.27 (17)
C8—C9—C10—C11	−179.30 (14)	C16—C15—N2—C7	−91.15 (19)
N3—C10—C11—C12	174.43 (16)	N1—C7—N3—C10	173.12 (13)
C9—C10—C11—C12	−1.5 (3)	N2—C7—N3—C10	−6.3 (2)
C10—C11—C12—C13	1.0 (3)	C9—C10—N3—C7	−1.1 (2)
C11—C12—C13—C14	0.3 (3)	C11—C10—N3—C7	−176.85 (14)

### Hydrogen-bond geometry (Å, °)

**Table:**

*D*—H···*A*	*D*—H	H···*A*	*D*···*A*	*D*—H···*A*
N1—H1A···O2	0.839 (18)	1.993 (19)	2.8017 (19)	161.8 (17)
O2—H2A···O1^i^	0.86 (2)	1.86 (2)	2.7180 (18)	174 (2)

Symmetry codes: (i) −*x*−1/2, *y*+1/2, −*z*+3/2.
